# Recruiting, Retaining and Engaging Men in Social Interventions: Lessons for Implementation Focusing on a Prison-based Parenting Intervention for Young Incarcerated Fathers

**DOI:** 10.1080/13575279.2017.1420034

**Published:** 2018-02-08

**Authors:** Katie Buston

**Affiliations:** ^a^ MRC/CSO Social and Public Health Sciences Unit, University of Glasgow, Glasgow, UK

**Keywords:** Prison, young fathers, parenting, interventions, engagement, implementation

## Abstract

Recruiting, retaining and engaging men in social interventions can be challenging. The focus of this paper is the successful implementation of a parenting programme for incarcerated fathers, delivered in a Young Offender Institution (YOI) in Scotland. Reasons for high levels of recruitment, retention and engagement are explored, with barriers identified. A qualitative design was employed using ethnographic approaches including participant observation of the programme, informal interactions, and formal interviews with programme participants, the facilitators and others involved in managing the programme. Framework analysis was conducted on the integrated data set. The prison as the setting for programme delivery was both an opportunity and a challenge. It enabled easy access to participants and required low levels of effort on their part to attend. The creation of a nurturing and safe environment within the prison classroom facilitated engagement: relationships between the facilitators and participants, and between the participants themselves were key to understanding high levels of retention and engagement. The most fundamental challenge to high engagement levels arose from clashes in embedded institutional ways of working, between the host institution and the organisation experienced in delivering such intervention work. This threatened to compromise trust between the participants and the facilitators. Whilst adding specifically to the very sparse literature on reaching incarcerated young fathers and engaging them in parenting work, the findings have transferability to other under-researched areas: the implementation of social interventions generally in the prison setting, and engaging marginalised fathers in parenting/family work in community settings. The paper highlights ways of overcoming some of the challenges faced.

## Introduction

Interventions for fathers are a recent growth area in family services; effectively representing fathers is a policy priority. There is now little doubt that fathers matter for the welfare of children and adults (Flouri & Buchanan, 2002, 2003, 2004; Flouri, Buchanan, & Bream, 2002; Kim, Kang, Yee, Shim, & Chung, 2016; Kroll, Carson, Redshaw, & Quigley, 2016). Programmes that potentially increase fathers’ involvement with their children are seen as an important complement to those that target mothers (Lundahl, Tollefson, Risser, & Lovejoy, 2008; Scourfield, Yi Cheung, & Macdonald, 2014). While there is established evidence that parenting interventions generally can be effective, the evidence base for parenting interventions which target fathers is much less developed and definitive. Studies which have been conducted, however, suggest that such parenting interventions may be effective in improving fathers’ involvement with their children (Bronte-Tinkew et al., 2008; Cowan, Cowan, Pruett, Pruett, & Wong, 2009; Magill-Evans, Harrison, Rempel, & Slater, 2006; Philip & O’Brien, 2012; Smith, Duggan, Bair-Merritt, & Cox, 2012).

Engagement in parenting interventions has been conceptualised as a sequential process, involving (a) initial take-up in response to recruitment procedures; (b) retention whereby participants return for the full course; (c) engagement with the delivery of the programme demonstrated by participation and interest (Lindsay et al., 2008; Moran, Ghate, & van der Merwe, 2004). There is some useful literature identifying the potential barriers parents face in engaging with parenting programmes, though there is little robust evidence as to “what works” (Axford, Lehtonen, Kaoukji, Tobin, & Berry, 2012; Katz, La Placa, & Hunter, 2007). The factors key to successful implementation appear to be whether parents can build up a trusting relationship with the front line service providers, and the degree to which parents feel they are in control of the help they are receiving.

Most studies around engagement in this area have focused on mothers (Panter-Brick et al., 2014). There is evidence that parenting programmes, and mainstream family services generally, fail to engage fathers (Maxwell, Scourfield, Featherstone, Holland, & Tolman, 2012; Scourfield et al., 2014). Bayley, Wallace, and Choudhry (2009) found that fathers tend to interpret the word “parent” as meaning mother when it is used on literature to advertise family services. Berlyn, Wise, and Soriano (2008) found that fathers were intimidated by the idea of accessing any sort of family support. In a study of young fathers’ experiences of the Family Nurse Partnership, Ferguson (2016) identified that their non-engagement arose from a combination of service delivery issues and from complexities around their own vulnerabilities and prior negative experiences as service users. Feeling excluded and/or judged are common experiences for young fathers, shaping their attitude to accessing family services (Fletcher & Visser, 2008; Ross, Church, Hill, Seaman, & Roberts, 2012), and seeking or receiving help is regarded as unmasculine (O’Brien, Hunt, & Hart, 2005).

There is also some work that suggests that parents living in poverty are more likely to be stressed and depressed, and this may hinder them from accessing parenting support (Katz, Corlyon, La Placa, & Hunter, 2007). In general, parents in areas of concentrated poverty often feel they lack the skills to become more involved.

Young parents, including fathers, also tend to be disadvantaged, in a number of ways which do not facilitate straightforward access to parenting support. Men who become fathers at a young age tend to have an accumulation of risk factors: low social class, early risk behaviour including sexual activity and substance use, mental health problems, lack of social support, and low educational attainment (Barlow et al., 2011; Buston, Parkes, Thomson, Wight, & Fenton, 2012). They tend to know little about child development or effective parenting skills (Barlow et al., 2011).

Being incarcerated exacerbates disadvantage still further, with any contact the father has with his child(ren) compromised by the period of his incarceration (Kazura, 2001; Nurse, 2001). Around one in four incarcerated young offenders in the UK are estimated to be actual or expectant fathers (Macmillan, 2005). There is a clear need for parenting interventions for young offender fathers to help them fulfil their roles as fathers, given the multi-faceted nature of their disadvantage, and to improve outcomes for their children (Lundahl et al., 2008) and themselves (Barlow et al., 2011). This need has been recognised and parenting intervention work has, indeed, been delivered in Young Offender Institutions (YOIs) in the UK for around 25 years (Buston et al., 2012), but has been characterised by patchiness and non-sustainability. There are few published process evaluations of parenting programmes in prisons (Miller et al., 2014), and none that the author is aware of in relation to interventions for young incarcerated fathers. A review of family interventions more broadly in the prison environment concluded that there were problems delivering such interventions in this setting because of: limited engagement with families, high participant drop-out rates, prisoner concerns about confidentiality, practical barriers such as lack of suitable rooms, and low trust between prisoners and staff (Roberts et al., 2016).

## Objectives

To contribute to filling the knowledge gap in relation to marginalised men and family interventions, and to add to the sparse literature on understanding implementation of social interventions in the prison setting, this paper reports results from a process evaluation of a prison-based parenting programme for incarcerated young fathers in a Young Offender Institution (YOI) in Scotland, UK during 2015. Recruitment, retention and engagement are the focus; facilitating and constraining factors are identified. The concluding discussion focuses on the prison as a setting for such work, highlighting specific opportunities and challenges encountered by this prison-based programme.

## Methods

In early 2014 the Scottish Prison Service (SPS) tendered for a third sector organisation to develop parenting services within HMP YOI Polmont, Scotland’s only YOI. At that time, and for the duration of the study, it had a capacity of just over 800 male prisoners, aged 16–21 years. Run by the SPS, strategic decisions around the institution, as is the case for all the SPS establishments, are made in conjunction with the Scottish Government. Contributing to the new vision for the prison service, which is increasingly expanding beyond protecting the public and reducing reoffending (Scottish Prison Service, 2013), the strategy for young offenders is to develop the institution as a learning environment. A key development area within this is Parenting and Families. Barnardo’s Scotland, a charitable organisation which works with vulnerable children, young people and their families to transform the children’s lives, won the tender to develop Parenting Services. Two Parenting Officers, Prison Officers employed by SPS, were recruited to facilitate this development and to co-deliver the core parenting intervention work.

In June 2014 Barnardo’s Scotland approached the author to evaluate early implementation of the core parenting programme, with the aim that results would feed into the development of the programme and to future delivery. *Parenting Matters* (an established parenting course delivered for over 20 years, largely in Northern Ireland) was used as a foundation for the programme, adapted for the incarcerated young fathers within HMP YOI Polmont. The resultant *Being a Young Dad* programme was delivered by the Barnardo’s Scotland facilitator and one of the two Parenting Officers over a full day a week for 10 consecutive weeks, with a condensed six-week version available for those men due to be released within these 10 weeks. It included information, skills and more reflectively based sessions. Sessions included: “what children need from parents”, “attachment”, “self-esteem”, “the importance of play”, “positive disciplining”, “behaviour management”, and “budgeting”. The Parenting Officers had also recruited a Peer Mentor from the group of long-term prisoners who had attended one of the first programme deliveries. His role was to help the Parenting Officers recruit fathers for future groups, and to reiterate key learning messages as well to help with practical tasks.

The programme was voluntary and open to fathers who, in theory if not always in practice, had access to their child(ren), or who had the prospect of such access. The 10-week programme included three additional and enhanced family visits, including a final celebratory visit, incorporating an award ceremony which marked the end of the programme.

The primary aim of *Being a Young Dad* was to help young fathers understand the positive role they could play in their child’s life, if they chose to do so. Key to the programme theory was the development of a positive relationship between participants and facilitators, and amongst the participants themselves. It was posited that if this could be achieved the young men would feel more relaxed and supported and would therefore engage to a greater degree, and actively participate in the programme, setting the context for behavioural change. Through the process of sharing and reflecting on their experiences it was theorised that they would realise the importance of the role of father, and this would motivate them to change, drawing on other skills taught during the course. As well as working through the formal content of sessions, the atmosphere created within the classroom session was regarded by Barnardo’s Scotland as key. Informal discussion, playing board games, and generally relaxing together were essential components of the course, alongside the traditional worksheet, materials on DVD, and directed discussions. Barnardo’s Scotland requested a kettle and fridge for the classroom, and the facilitator provided milk and biscuits at each session in order that the young men could help themselves to refreshments. Money was also spent on resources for the programme, including games such as Monopoly, a stock of occasion cards they could send to loved ones, a radio for background music, and bean bags for relaxation.

After receiving permission from both the SPS Research and Ethics Committee and Glasgow University’s Social Science Ethics Committee (400130248), the process evaluation commenced, comprising a qualitative study, using multiple methods, of the programme implementation:

1. Participant observation of delivery of all the parenting sessions of the programme (*n* = 10 sessions), and some of the sessions (*n* = 4 sessions) of another group who were undertaking the condensed version of the programme over six weeks. Each “session” comprised a full day, divided into a morning and an afternoon with the men returning to the halls at lunchtime. Between February and April 2015 over 100 hours was spent by the author in the parenting classroom, observing, and participating in, the programme delivery and “hanging around” between sessions talking to the staff and the young men.
2. Attendance at family visits (*n* = 3) in the visiting room, providing an opportunity to observe some of the young men interacting with their child(ren) and their partners, or ex-partners, during the two-hour visit period.
3. In-depth interviews (*n* = 6) with the young men who had completed the programme and who were still in the prison six weeks after its completion. The topic guide for these interviews included exploration of their motivation for attending the course, and of their views around how they were approached to participate in it; and how they felt about the programme itself exploring aspects around their participation and engagement.
4. An in-depth interview (*n* = 1) and numerous conversations with Barnardo’s managers and other staff before, during, and after the fieldwork period.
5. Prolonged contact with those involved in delivery during the analysis period, with occasional discussions (in person and by email) about emerging results, including in the context of continuing delivery of the programme following the period of fieldwork.
6. Collection of copies of the end of programme reports written by one of the facilitators for each man in her case load (*n* = 8). These commented systematically on attendance, the sessions covered and the individual men’s engagement with the course and with particular aspects of it.
7. Collection of written materials about the programme and its delivery, passed on by the facilitators and those within Barnardo’s Scotland who introduced the programme to HMP YOI Polmont.

For (1) and (2) and the conversations referred to in (4), detailed ethnographic fieldnotes were written on leaving the prison, aided by the notes taken during the day, often in code, as an aide memoire of the day’s structure. These included notes on what was delivered, by whom and how, and who was there and to what extent did they participate and how, including who said what—formally and in conversation with the author, as well as overheard. For (3) and (4) the in-depth interviews were recorded and transcribed with participants’ permission. For conversations which took place with staff outside the prison—in person, by email or on the telephone—pertinent comments were recorded in a Word file which was added to the integrated data set.

All of these data sources have been analysed in an integrated way for this paper (Saldana, 2013). Barnardo’s were keen to learn how the intervention was being implemented: what was being delivered, how was it being delivered, and how were the participants receiving it (that is reacting to it, and engaging with it). The data were coded by the author, initially in a descriptive way followed by more explanatory work, in order to answer these questions. “Recruitment”, “retention” and “engagement” were coding categories, and within these “nodes”, facilitating and constraining factors were identified, for example “trust”, “institutional practices”, and “practical issues”. Framework analysis was used with examples charted in order to manage and summarise the voluminous data (Gale, Heath, Cameron, Rashid, & Redwood, 2013; Ritchie & Lewis, 2003).

Where quotes from young men are presented, below, a pseudonym is used. Pseudonyms have also been used for the prison staff.

## Findings

Sixteen fathers participated in the two programme deliveries observed. These included those with long-term sentences, as well as those with shorter sentences and remand prisoners. The men were aged between 17 and 21 years, and had between one and three children. Some were still in a relationship with the mother of their child(ren) but most were not. Nearly all had some current contact with their child(ren).

### Recruitment

The primary way of identifying and approaching fathers within the YOI in order to recruit them was through the Parenting Officers, and the Peer Mentor, attending Induction. Induction is when new prisoners are introduced to the rules, guidelines, and processes of the prison, including how visiting works and what job opportunities exist. They talked to the new arrivals during this session, and asked them whether they were fathers, or expectant fathers. The Parenting Officers and Peer Mentor then followed up those who had identified themselves as such, checked that they were eligible to receive the core programme, and visited their cells to tell them about what the core parenting programme involved, answering any questions, and inviting them along to the first session. The Parenting Officers estimate that, since they began delivering the programme around two and a half years ago, fewer than 5% of the men they have approached in this way said that they were not interested in attending. According to the Parenting Officers most of the small number who did decline did so because of the complexity of their relationship with the mother of their child and future prospects of developing a relationship with the child because of this. A small number of men were recruited through Addictions, and other, workers telling them about the programme during one-to-one sessions where their parenting was discussed.

### Retention

Nearly all of those men who attended the first session of the various deliveries of the programme that have been run to date, including all of those attending the deliveries observed for this study, went on to attend the rest of the programme. It was common, however, for individual men to miss two or three mornings or afternoons of the programme due to occurrences such as court appearances, health centre or social work appointments, or hall lockdown. For the shorter course observed, two men were liberated before the end of the course. Aside from these events beyond the control of the individual men, however, attendance was high. None of the men participating in the deliveries observed for this study intentionally missed particular mornings or afternoons because they decided not to attend; all appeared committed to the programme, none dropped out. Commitment to attending was very high.

### Engagement

Furthermore, all of the men observed for this study appeared to engage with the programme on a consistent basis, though actively participating in different ways and to varying extents. Timmy, for example, was quiet, rarely contributing verbally, but was on task with most of the activities, including filling in worksheets and creating a book for his baby. Jimmy, on the other hand, verbally contributed frequently, often making jokes. He liked to “banter” with the facilitators, with this sometimes crossing the line into aggression. His contributions were, however, always relevant to the content of the session, and could usually be constructively used by the facilitators to emphasise relevant learning or to provoke further discussion amongst the class. Even Dylan, who had a history of addiction and problems concentrating, reported enjoying the programme and actively contributed on a regular basis. It had taken a few weeks for him to feel comfortable:

I did not really say much at first, man, just liked to know what other people were saying and all that, and then I started speaking a couple of weeks later. But aye, it [the programme] was worth it, definitely.

Engagement was high, and did not vary greatly from week to week or topic to topic, or even from man to man in terms of level, if not style, of engagement. Towards the end of the 10-week programme, there were several discussions initiated by the men around why they were not able to begin the programme again once they finished that programme. Most of them expressed a desire to “keep on coming along”. Indeed, the facilitators set up a “keeping in touch” group for which take-up has been high according to post-fieldwork reports from the facilitators to the author.

### Factors facilitating successful implementation

#### Relationship between facilitators and participants

The positive relationships between the facilitators and the participants was regarded by Barnardo’s Scotland as a key potential mechanism for change. As a Barnardo’s manager said:

If we can do that for those dads, then they can do that for their children ’cause they know that it feels good to have somebody be positive. If we can help someone to know how it feels to have somebody else thinking about you, then can they open up something for their little one.

It was regarded as essential that the programme was delivered in a conducive context where the facilitators demonstrated respect for the participants of the course and built up nurturing relationships.

The two Parenting Officers recruited to deliver the programme were very different in their demographic profile and in their manner and style. They were both Prison Officers with many years’ experience of working with the men in the halls; one had worked for the SPS since leaving university and the other had come to the SPS after being in the armed forces. One was male, the other female; one had children and a spouse, the other had a partner but no children. One was very open and willing to discuss personal issues with the young men, the other was much more private though did share some specific examples relating to parenthood. Both used humour in delivering the programme and were adept at managing the young men’s behaviour, keeping them focused on the task at hand, whilst being respectful to them. While it was clear who was in charge and clear boundaries were maintained, the Parenting Officers interacted with them on first name terms and chatted with them about many aspects of their lives, treating them as much more than simply “prisoners”.

One Parenting Officer expressed frustration on one or more occasions around the “heavy handedness” of other Prison Officers in their treatment of the young men around their participation in the parenting intervention. For example, it was observed that during a family visit one of the men was taken away from his partner and children as it was suspected his partner had handed him drugs. The Parenting Officer felt that there was no need to have “marched him off” to be searched when his child was present, and that the situation—whilst it had to be addressed—could have been dealt with in a subtle way. While the two Parenting Officers were very clearly inculcated into the culture of the YOI, having worked as Prison Officers for many years, they explicitly recognised (and it was implicit in their observed behaviour) that a slightly different relationship was required in their role as Parenting Officers, and were protective of the programme participants when they felt that “normal prison culture” was impinging on the more caring ethos surrounding the parenting programme.

The ways in which the facilitators endeavoured to build this different sort of relationship with the participants, winning their trust, included calling them by their first names, and introducing themselves by their first names; offering them cups of coffee and snacks; giving them positive personal feedback; having quite lengthy conversations with them about parts of their lives which had nothing to do with their status as prisoners; and sharing some aspects of their own lives with the men. Much of this had been encouraged by Barnardo’s when the partnership was first initiated. As one of the Barnardo’s staff said:

it’s that relation of currency, and I think perhaps in the hall mentalities you [the Prison Officer] don’t tell them [the men] anything, but I think you have to. If you’re going to be part of somebody’s life and meeting their child and their partner, and they’re letting you in, you need to give something.

From what was observed, for this study several months after the working partnership between the SPS and Barnardo’s had begun, the Parenting Officers had great respect for the men, even when individual men were exhibiting challenging behaviour as happened from time to time.

For the Barnardo’s facilitator, it was perhaps more “natural” to develop such a respectful relationship with the young men than it was for these Parenting Officers who had worked in the halls of prisons for many years. Although this was the first time she had delivered a programme within a prison, and she had undertaken much more work with mothers than with fathers, she was very experienced in delivering parenting work to vulnerable populations, and well versed in what she called “reaching out to the most disadvantaged families”, a central tenet of Barnardo’s work.

The men did notice, and appreciate, efforts to build relationships in these ways. As Dino said:

see in the halls they’re [Prison Officers] not like that, but up here, aye. Tom and Heather are sound man, they’re all right.

Harry said:

Everyone that was part of the course was good people … if they weren’t I wouldn’t have paid any attention, I wouldn’t have cared, I wouldn’t have bothered with it. Respect goes a long way.

#### Relationships between the men

In their conscious quest to create respectful caring relationships with the men, it was hoped that a by-product would be the men developing caring relationships with each other as the parenting classroom became a place where all within it would listen to each other, respect would prevail, and it would be free of aggression and conflict and safe for all to express their feelings and thoughts.

The opening session included the men setting ground rules. They were encouraged to come up with some rules that would create a safe and caring atmosphere within the classroom: what is said in the room is confidential; no judging each other; no *personal* banter; and respect each other.

When the Parenting Officers were aware that there had been conflict between individual men in the past they worked to try to ensure that this was left behind within the parenting classroom. For example, Saul and Jimmy had a history of conflict in their community, before imprisonment, but were in the same parenting group. The Parenting Officers did some conciliation work with them before the course began.

When racist, homophobic, sexist or similar remarks were made within the classroom, they were usually challenged, in a firm but respectful way, by the facilitators. Jimmy, for example, often made derogative remarks about his partner, the mother of his children. Over the weeks, it was observed that these were challenged not only by the facilitators, but by the other men. When Dylan made homophobic remarks, with violent connotations, his statements were deemed unacceptable and were unpacked by the facilitators. They talked him through how such comments may make others feel. Barnardo’s staff had, initially, had concerns that challenging the men in this way may not be something the Parenting Officers were easily able to do. A manager reported that Barnardo’s Equality and Diversity training had been undertaken by the Parenting Officers, in addition to SPS training, and from what was observed the Parenting Officers appeared to be comfortable with such challenging.

For both the groups observed, there was little or no conflict between the men, though the amount of “banter”—which could be challenging for the facilitators to manage—varied depending on which particular individuals were there for each session. Generally, it was observed that the bigger the group, the more high spirited it was, but even at the times of peak liveliness, the young men managed to stay on task and to put their interactions with each other aside to focus on the session itself. However, since this fieldwork was undertaken, the facilitators have reported that there have been a small number of groups which have failed to work well together, and where engagement has been compromised as a result.

#### The classroom climate

The classroom climate is a product of the relationships discussed above, as well as being shaped by things such as the aesthetic environment, discussed below. Buston (2018) discusses in detail how a caring, sharing climate was created in the deliveries observed, a climate which allowed the men to show their softer side in the parenting classroom. Being a father and showing emotion in relation to one’s children and partner was an acceptable masculine identity within the classroom.

As well as the work done by the facilitators to foster conducive relationships between themselves and the men, and between the men, there were also other nurturing efforts made. The Barnardo’s facilitator brought in milk each day, and fruit or biscuits, and the classroom was stocked with coffee, tea and juice. If the men talked about missing particular items of food, she would sometimes buy these. As Dino said:

we do appreciate that [provision of food and drink] because we don’t get decent, a lot of folk don’t get a lot of money sent in so they don’t go and buy a bag of Nescafe ’cause it’s 4.50, a lot of the boys are like that “no, I can’t pay that for a bag”, so they appreciate it. And biscuits, you don’t get biscuits like that in here. Feel looked after, aye.

The room itself was light and bright, there were beanbags to sit on as well as the more traditional tables and chairs, and at times the radio was switched on to provide background music. One of the Barnardo’s managers described it:

they’ve got this amazing new room. It’s beautiful, this whole activities building is just a different world. It’s brightly coloured, just bright colours everywhere, like nice images. It’s so lovely and apparently the dads all said, ’cause they were in it last week I think, and they were like “oh, it’s like a college. It’s like being in a college”. Which is what you want. You want them to have a different sense [there than in the rest of the prison], a sense of learning.

#### Content and methods

*What* was delivered, and *how* it was delivered, also facilitated ongoing engagement. Observations suggested the men were open to learning about a variety of topics, and liked to discuss, participate in quizzes, and undertake practical exercises such as cooking and making cards. This was corroborated by the interview data with the young men. The facilitators appeared to be very good at gauging if boredom was rising, and engagement was waning, and were flexible in their delivery of particular segments, knowing when to cease or change pace or direction. It was observed that when concentration flagged, board games were brought out or there was informal discussion over coffee. The Parenting Officer and Barnardo’s facilitator were, overall, much more successful in engaging the men than outside facilitators, though this varied. The men were fascinated by a visitor from the Fire Brigade, for example, who talked about fire safety in a humorous, animated way, but were bored—the observations and interview data suggest—by a member of the prison health staff who came in to talk about passive smoking and the dangers of drugs. The manner of the visitor, and the extent to which s/he appeared to be able to relate to the young men, had a bigger impact than the content itself.

The men reported enjoying arts and crafts activities, this was evident from observations also. They usually had an ongoing craft project, for example making a Mother’s Day card or a book for their child. Often at the end of a morning or afternoon, when they were tired of doing other things, these were brought out. During the sessions which were observed, some of the men asked that they take the items back to their cells to work on. A mix of activities during each session worked best: a discussion session followed by a game; or skills-based work followed by some arts and crafts.

#### The prison context

Both the financial and opportunity cost of, and the effort involved in, attending an intervention such as this must be considered. If this was offered in the community, the young fathers might miss valuable leisure time if they were to attend. It might cost them money to get to the venue by bus or taxi, and require effort to plan and get themselves there at the same time each week. In the prison, however, there is little to do during the day. Most of those on the programme reported that they would have been in their cell, trying to fill their time, if they were not at the parenting session. The men needed to make very little effort to attend the classes; all that was required was that they wait for the ‘prison route’ (when prisoners are escorted by Prison Officers from one part of the prison to another) to take them from their hall to the parenting classroom. From the point of view of those implementing the course, therefore, the men were very accessible. The facilitators reported that the men could be approached at induction or in their cells where they could be asked to identify themselves as fathers, and that they also had access to other sources of information about the men's fatherhood status. These young men, imprisoned in the Scottish context, were drug and alcohol free when incarcerated; many would not be in the community.

In terms of the men’s motivation, some of them mentioned that, when they had first been approached about the programme at least, they had thought their attendance might “count for something” with social work, or in terms of an early release. None, however, seemed to then continue to attend simply so it looked good in this way. Timmy, for example, who had not yet secured access to his baby and reported desperately wanting this contact, explained that his prime motivation for attending the course was to show social work that he really did want to be a father to his child; he felt this commitment “proved” this in a way he was not able to do with his words alone. His reports, and what was observed, suggested that he wanted to learn during the programme so that when/if he did get access he would know how to look after and interact with his child:

see as long as it’s going to get social work off my back, to make them see that I’m not just putting a front on ’cause I’m in the jail doing this. I’m doing it just to show them that I am trying, I *do* want to be a dad.

Some of the men also talked about a general desire to make themselves better people during their prison sentence, which may also have been the prime motivation for first attending for some. Overall, though, there was a sense amongst the men that this programme might actually help them to be better fathers, something that they all said they wanted. Many of them had had traumatic upbringings; this often became increasingly apparent as the programme progressed, and they wanted to know how to provide the loving and caring environment for their child(ren) that they had not had.

### Barriers to successful implementation

There were barriers and challenges which compromised, or threatened to compromise, individual men’s ongoing engagement from time to time. Barnardo’s had had to undertake considerable amounts of “behind the scenes” work as a foundation for the intervention delivery in the prison. Introducing “new ways” of working to the YOI was challenging. Sometimes these new ways of working, seen by Barnardo’s as a necessary foundation for implementation of the intervention, were threatened. For example, one of the Barnardo’s staff members described the resistance of prison staff when the facilitator bought one of the participants a birthday card and cake on his 21st birthday. She felt that while the Barnardo’s staff member saw it as an “obvious” aspect of nurturing, core to the intervention, prison staff saw it as “alien”. As one of Barnardo’s managers said:

It’s a very difficult environment to do this in. Not because people are deliberately obstructive, people just don’t work in that way … I don’t think it’s a malicious thing … . It’s just that they don’t know [this way of working]. Stuff that we [Barnardo’s] just do without thinking and I think “oh, they’ll just know how to do that”, and they don’t.

The Barnardo’s staff were very reflective about their ways of working, and how the ethos of Barnardo’s as an organisation was very different to that of SPS. Interviews and conversations with these staff described the groundwork undertaken at the early stages of intervention delivery. During the fieldwork, however, there were several instances where organisational value clashes became apparent. These usually stemmed from differences relating to how the young men were viewed, with the ethos of Barnardo’s dictating they should be treated as equals, and valued in an unconditional way, but the institutional mores of the prison service not always facilitating this to the extent Barnardo’s would find acceptable in order to maintain the trust necessary to fully engage the men on an ongoing basis in the intervention work.

There were also social work and other issues around the men’s status as fathers which were outside the control of those delivering the programme, but that sometimes compromised the men’s willingness to engage. The men sometimes felt frustrated with the facilitators if social work meetings around access were slow to organise, or decisions did not go their way, and were then less amenable to participating in classroom sessions. During one of the observed family visits Timmy was expecting his ex-girlfriend and baby but they did not arrive. A Prison Officer had not been available to escort Timmy back to the halls once it was apparent that his child was not coming, so he had had to sit with the other men and their families as they interacted. He was visibly upset during this time. The Parenting Officers reported how they had to undertake one-to-one counselling with Timmy following this to ensure that he felt able to complete the rest of the course following this incident. Interviews and conversations suggested that he, the facilitators, and the other men who had received visits that day felt very uncomfortable with him having to sit through the visits from other partners and children.

There were groups, not observed during the fieldwork but talked about by the facilitators, where members failed to work well together, making delivery and the facilitation of engagement difficult. This was usually, it was reported, down to the presence of one disruptive individual. Many of the young men in the YOI had problems concentrating and/or reading and writing, but the responses to the programme by those with the most extreme difficulties in these areas have sometimes made it difficult for their class-mates to engage. Barnardo’s are very clear that these men, amongst the most vulnerable in the prison, should not be excluded from the group, but it is recognised that these individuals can compromise others’ engagement. Staff report working on solutions to ensure that these men can be included, and feel able to engage, and do not hijack the experiences of others.

## Discussion and conclusions

The paper has focused on the recruitment, retention and engagement of young incarcerated men in a prison-based parenting intervention for fathers, elucidating processes of implementation and identifying facilitating factors and barriers. Qualitative data collection has utilised a range of methods to collect data from, and with, a number of incarcerated men and staff involved in managing and delivering the intervention, over a prolonged period of time, and spanning different deliveries of the same programme delivered by different facilitators. Framework analysis was undertaken on the integrated data set. Whilst adding specifically to the very sparse literature on engaging incarcerated young fathers in parenting work, the findings have transferability to other under-researched areas. They will be particularly useful for those working in the prison setting implementing any social intervention with inmates, and also have utility for those working in the community implementing parenting and family work with marginalised young fathers.

This intervention is an example of a parenting intervention which successfully recruited, retained and engaged fathers. Furthermore, it successfully recruited, retained and engaged marginalised, vulnerable, traditionally “hard-to-reach” fathers. Why was it successful?

First, trust and respect were key in motivating engagement, concurring with existing literature (Axford et al., 2012; Katz et al., 2007; Pfitzner, Humphreys, & Hegarty, 2017). Those delivering the programme invested time and effort in building relationships with parents. Particularly in the prison environment, where there is a general mistrust towards prison service staff, the Parenting Officers, in their prison uniforms and as SPS representatives, were successful in winning the young men’s trust through their manner. The involvement of an outside facilitator, in civilian clothes and clearly not employed by the prison service, probably helped. Her skills in delivering parenting work and forming such trusting relationships over a number of years were key.

Second, the practical aspects involved facilitated engagement (Pfitzner et al., 2017). A programme delivered, almost literally, on the doorstep of the men’s cells with no financial cost and little opportunity cost involved in attendance will attract and retain the men more than one where costs are high in these terms.

Third, that this programme focused specifically on fathers, in the context of an all male prison, seems important, though this is hard to draw conclusions around as there is no comparison of a mixed sex prison parenting programme. Certainly these men seemed to find commonalities with each other. It was implicit in much of their interaction that they came from similar communities, had similar backgrounds, and were of similar socio-economic status. They were all at the same life stage and their children were all very young. Furthermore, there was an understanding amongst them that they had taken a similar path in life, ending, for now, there in the jail, and they appeared to have similar knowledge, attitudes and behaviours with regard to parenting as well as other topics. As young fathers, they sometimes talked about how they perceived they were being judged as deficient in some ways by others, appearing to share an identity as fathers who cared but who had to overcome some barriers in order to be seen as “proper parents”. They generally saw their role as fathers as being distinct from that of mothers. The homogeneity of the group in all of these respects meant it was fairly straightforward for the facilitators to ensure that content was relevant and of interest to all members of the group, a characteristic of effective social interventions (Bronte-Tinkew et al., 2008). Indeed, this homogeneity facilitated high levels of engagement both because group members were able to bond together and because approaches and content could be targeted to the needs and interests of all the men. Available evidence does indicate that the involvement of both parents in interventions is the optimal way forward (Lundahl et al., 2008; Ramchandani & Iles, 2014) but opportunities such as this where groups of otherwise hard-to-reach men can be successfully involved in and engaged with targeted parenting work should be grasped.

Fourth, the men’s desires to be “good fathers” should be noted. Young incarcerated men appear to aspire to being caring fathers: warm, sensitive, attentive, protective of their child, and spending time with and supporting him/her (Buston, 2010; Buston, O’Brien, & Maxwell, in preparation). All of the fathers participating in the two deliveries observed for this study said that they wanted to be good fathers. Nearly all of them reported unhappy childhoods and wanted to be there for their child(ren) in the way their biological father had not been (Buston et al., in preparation). Furthermore, most recognised that the way they had been fathered was a potential barrier to them fathering in positive ways. They wanted to be good fathers, and they recognised that attending this parenting course could be helpful in facilitating this.

This study was a process evaluation. It did not focus on outcomes, instead seeking to examine how the intervention was implemented. It was very much formative, and exploratory, work, designed to be able to feedback to Barnardo’s on the initial deliveries of the programme with this population, suggesting mechanisms which might lead to it being effective across the men, while identifying barriers and facilitating factors to it being delivered, and received, as intended. The next step would be to conduct a rigorous outcome evaluation. The results reported in this paper, and those to be reported elsewhere (Buston, in press; Buston et al., in preparation) will form a solid foundation for this work.

Recent work has noted that engaging with fathers is one of the least well-explored and articulated aspects of parenting interventions (La Placa & Corlyon, 2014; Panter-Brick et al., 2014), and, indeed, of family services generally (Scourfield et al., 2014). This paper contributes to better understanding some of the mechanisms which facilitate engagement. Findings highlight the need to look beyond content in understanding why any parenting programme, in the prison or in the community, might successfully engage its participants in a sustained way. These fathers very much appreciated the classroom context in which the programme was delivered; a caring, sharing, nurturing ethos within the parenting classroom will facilitate engagement amongst similar groups of marginalised young fathers within the community also, and there are lessons to be learned for engaging fathers in family services more generally. The work supports assertions that particular parents are not, by dint of who they are, necessarily “hard-to-reach” but instead it may be that particular interventions and services are hard [for them] to access (Davies, 2016). The prison is an opportunity for delivering parenting programmes to incarcerated fathers, an opportunity that should be grasped as it can be a highly conducive setting for such work, albeit with institutional challenges, some of which threatened to compromise aspects of engagement for this programme (Miller et al., 2014).
